# The Book World of Medicine and Science

**Published:** 1900-05-05

**Authors:** 


### The Book World of Medicine and Science.

Pye's Surgical Handicraft. A Manual of Surgical Manipulation, Minor Surgery, and other matters connected with the Work of House Surgeons and Surgical Dressers. Fourth edition, revised and edited by Bertram M. H. Rogers, B.A., M.D., B.Ch.Oxon. (Bristol: John Wright and Co. 1900. Price 10s. 6d.)

This book is so well known that it is not necessary to say much on the appearance of its fourth edition beyond congratulating its editor on the judicious manner in which he has introduced new matter and eliminated old in such a way as to bring the work up to the requirements of modern surgery, which it need hardly be said are very different from those even of a few years ago. As was well said by the original author, the late Mr. Pye, "However scientific surgery may become, and however largely surgeons may find it necessary to concern themselves with higher problems, the purely manipulative side of their work must never be disregarded, for if this work is to be well done 'chirurgery can never be false to its etymology, χειρ εργον, will never cease, that is, to be a skilled labour, nor will surgeons ever cease to be handicraftsmen.'" It is, then, with the manipulative side of his work, with the handicraft of surgery, the correct and perfect performance of which is a matter of such importance to dressers and house surgeons, that this book more especially deals.

It commences with a good chapter on hæmorrhage and the various means to be adopted for its arrest. All these are well described. A full account is also given of tranfusion of blood, both mediate and immediate transfusion being described, after which saline transfusion is mentioned, which, it is stated, is "almost if not quite as efficacious as blood transfusion," a sentence which perhaps hardly fully expresses the position now occupied by the intravenous infusion of saline solution. It is worth noting that the temperature of the water which is to be added to the salt is given as being from 98 to 99 deg. Fahr., rather low, we think, especially as according to the method described the water at this temperature is to be added to the salt kept ready in a bottle which will hold two pints, so that some loss of heat has to be reckoned with. Curiously, although the injection of several pints of warm water into the rectum is mentioned as an alternative method, no reference is made to the plan of infusing saline solution into the subcutaneous tissues. A good and complete account is given of bandages, splints, and apparatus, and of the treatment of fractures and dislocations. A good deal that is new has been introduced into the chapter dealing with the antiseptic dressing of wounds, while much that has become out of date has been omitted. Some people may say that the precautions recommended savour of simplicity. It is stated that "to purify the hands they should be well scrubbed with a nail brush and soap, particular attention being given to the nails, and then soaked for a few minutes in carbolic acid lotion (1 in 30, or weaker) or perchloride of mercury lotion," and it is added, "Very elaborate methods are given in some works on surgery, but we believe a good scrub with soap and water, with a soak in lotion, is quite sufficient." Perhaps; but we are surprised to see no mention of the use of either turpentine, or spirit, or ether to assist in the removal of the grease from the skin of either the surgeon or the patient. The strong point of the work lies in its description of manipulative procedures, minor surgery, and the hundred and one things which a house surgeon has to do. All these are thoroughly well given, and as a dressers' handbook "Surgical Handicraft" is sure to prove useful, concentrating as it does into small compass a good deal of information which without it would have to be sought through many pages in larger treatises.

Primer of the Physiological Action of Alcohol. By Edwin J. Norris, M.R.C.S., L.R.C.P., Lecturer on Physiology and Hygiene, Municipal Technical Institute, Portsmouth. Introduction by Dr. Clifford. (London: Swan Sonnenschein and Co., Limited. 1900. Price 1s. net.)

The author of this little elementary text-book has presented to the reader in concise form "a simple but accurate account of the action of alcohol" upon the various organs of the body. The primer is intended for use in those schools where "Temperance Physiology" forms one of the subjects of the curriculum, and is, we believe, the first book of its kind. Our colonies make proper provision for the teaching of this subject to the young in the State-paid schools, and rightly recognise the necessity of impressing a knowledge of alcohol and its action at an impressionable age. The text is copiously illustrated, the illustrations reaching a very fair standard of merit. We notice as a misprint that, on page 9, "Yes" ought to be "Yeo." We congratulate the author on the pithy manner in which he puts his statements, and upon his power of condensing matter and, at the same time, giving it in an interested and connected form.

Golden Rules of Physiology. By I. Walker Hall, M.B., Ch.B.Vict., Senior Demonstrator in Physiology, The Owens College, Victoria University, and J. A. Menzies, M.D., C.M.Edin. (Bristol: John Wright and Co. 1900. Pp. 80. Price 1s.)

This booklet, which forms the sixth of the "Golden Series," is, we are informed in the preface, "intended for the use of students who have already attended lectures and read a text-book, and who are on the threshold of examination," as a stimulation to thought and memory. The contents are arranged under alphabetical heads for facility of reference, but no attempt is made at the systematic physiological classification of the rules. This series gives valuable help to those students who use it in the way it is intended, but we fear there are some who may place too great reliance on these little books, and thus frustrate the authors' purpose; we have, however, nothing but praise to accord to this little book, which fully maintains the standard of the previous numbers of the series.

### NOTES ON BOOKS.

The Matriculation Directory, January, 1900 (W. B. Clive), is a handy little book, which gives the reader full information as to all details connected with the London matriculation examination. The University regulations, the choice of text-books, the general and special subjects, the papers set at the last examination in January, with model answers in the subjects usually taken up, form the contents. The intending student cannot do better than follow the advice given in the article on "Text-Books." This key to the matriculation is already so well known that it needs no further mention at our hands.

Maps are essential to knowledge, and are especially wanted, along with the morning paper, at the breakfast table. Still a great sprawling map is somewhat of a nuisance. It upsets the coffee, sticks in the marmalade, and makes itself generally objectionable. These little difficulties, however, are all overcome in Bacon's War Atlas, in which the geography of South Africa is depicted in a series of large-scale maps opening bookwise. The whole arrangement is very convenient, and certainly is a good shillingsworth.

